# Role of NHE1 in Nociception

**DOI:** 10.1155/2013/217864

**Published:** 2013-01-30

**Authors:** Jorge Elías Torres-López, Crystell Guadalupe Guzmán-Priego, Héctor Isaac Rocha-González, Vinicio Granados-Soto

**Affiliations:** ^1^Centro de Investigación y Posgrado y División Académica de Ciencias de la Salud, Universidad Juárez Autónoma de Tabasco, 86040 Villahermosa, TAB, Mexico; ^2^Hospital Regional de Alta Especialidad “Dr. Juan Graham Casasús”, 86126 Villahermosa, TAB, Mexico; ^3^Sección de Estudios de Posgrado e Investigación, Escuela Superior de Medicina, Instituto Politécnico Nacional, 11340 México, DF, Mexico; ^4^Departamento de Farmacobiología, Centro de Investigación y de Estudios Avanzados (Cinvestav), Sede Sur, Calzada Tenorios 235, Colonia Granjas Coapa, 14330 México, DF, Mexico

## Abstract

Intracellular pH is a fundamental parameter to cell function that requires tight homeostasis. In the absence of any regulation, excessive acidification of the cytosol would have the tendency to produce cellular damage. Mammalian Na^+^/H^+^ exchangers (NHEs) are electroneutral Na^+^-dependent proteins that exchange extracellular Na^+^ for intracellular H^+^. To date, there are 9 identified NHE isoforms where NHE1 is the most ubiquitous member, known as the housekeeping exchanger. NHE1 seems to have a protective role in the ischemia-reperfusion injury and other inflammatory diseases. In nociception, NHE1 is found in neurons along nociceptive pathways, and its pharmacological inhibition increases nociceptive behavior in acute pain models at peripheral and central levels. Electrophysiological studies also show that NHE modulates electrical activity of primary nociceptive terminals. However, its role in neuropathic pain still remains controversial. In humans, NHE1 may be responsible for inflammatory bowel diseases since its expression is reduced in Crohn's disease and ulcerative colitis. The purpose of this work is to provide a review of the evidence about participation of NHE1 in the nociceptive processing.

## 1. Introduction

Intracellular pH (pHi) is a fundamental parameter to cell function that requires tight homeostasis [1]. In the absence of any regulation, the cytosol would have the tendency to become acidified due to the continuous buildup of metabolic acid (H^+^) equivalents [2, 3]. Cells have developed means to raise cytosolic pH, guarding against dangerous acidification. Regulation of pHi comprises several processes such as cytosolic H^+^ buffering, H^+^ sequestration into cellular organelles, and transmembrane movement of acid equivalents [1, 3, 4]. Cells regulate rapid and localized pH swings by their intrinsic pH buffering capacity which is provided by several intracellular weak acids and bases. Moreover, cells regulate pH through the bicarbonate (HCO_3_^−^) buffer system which combines with excess H^+^ ions to form carbonic acid [5]. Then, carbonic acid is transformed to carbon dioxide (CO_2_) by the enzyme carbonic anhydrase [5]. The total buffer capacity includes both components [1, 2, 4]. Although effective this buffering system has limited capacity to counteract continuous generation of H^+^ equivalents by metabolism, ongoing transport of ions that alter the pH (H^+^ and HCO_3_^−^), or the presence of diseases that contribute to extracellular acidification (inflammation, hypoxia, or ischemia). The mechanism of regulation of pHi carried out by transporters requires energy as H^+^ is transported against its electrochemical gradient. Thus, transporters use the inward Na^+^ gradient produced by the 3Na^+^/2K^+^-ATPase. Several proteins carry out this function being one of the most important Na^+^/H^+^ exchangers [1, 6, 7].

Mammalian Na^+^/H^+^ exchangers (NHEs) are electroneutral Na^+^-dependent proteins that exchange extracellular Na^+^ for intracellular H^+^ [8–10]. In animal cells, they are linked to a variety of physiological roles which include regulation of pHi and cell volume [6, 10–13]. Between the different NHE isoforms identified, NHE1 is the most ubiquitous member [9, 10]. In pathological conditions, the activity of NHE1 has been related to growth of some tumor cells [14]. Moreover, NHE1 has been proposed as a mediator of the myocardial damage that occurs after ischemia-reperfusion injury [15, 16]. However, recent studies with transgenic mouse models expressing elevated NHE1 levels have demonstrated less susceptibility to ischemia-reperfusion injury [17–19] suggesting that NHE1 plays a protective instead of noxious effect in this pathology. Besides its role in cancer and ischemia, recent evidence has shown the NHE1 plays an important role as a protective mechanism in nociceptive sensory neurons. The aim of this work is to provide a review of the role of NHE1 in the nociceptive processing.

### 1.1. NHE Family

The NHE system was first identified in 1977 [20]. Later (in 1988), NHE was cloned [21, 22] and started an explosion of research on these proteins. To date, there are 9 identified NHE (gene SLC9A) isoforms, NHE1 (gene SLC9A1) to NHE9 (gene SLC9A9) (Table 1). NHE1-5 are localized in plasma membrane whereas NHE6-9 are found in intracellular compartments. NHEs vary in their cation selectivity and localization. Regarding the latter, NHE1 is the most ubiquitous member, known as the “housekeeping” exchanger [10, 23]. NHE2 through NHE5 have a more limited tissue distribution. NHE2, NHE3, and NHE4 are expressed predominantly in the kidney and gastrointestinal tract [24–27] whereas NHE5 is predominantly expressed in the nervous system [28, 29]. The organellar isoforms include NHE6 (mitochondria and endoplasmic reticulum membrane), NHE7 (recycling endosomes), NHE8 (trans-Golgi network), and NHE9 (recycling endosomes) [30, 31].

### 1.2. NHE Structure

Human NHE proteins have between 645 and 898 amino acids characterized by two domains: the N-terminus transmembrane transport domain and the C-terminus regulation domain [10]. The N-terminus is highly homologous among isoforms [32]. Although controversial, NHEs have 12 putative encoded transmembrane spanning domains with both N- and C-terminus located in the intracellular side [23, 32].

There are suggestions that NHEs have a cleaved signal peptide and 11 functional transmembrane spanning domains, an extracellular N-terminus, and an intracellular C-terminus [27]. However, it could be confirmed by further studies. Transmembrane domains 4 and 9 are involved in the sensitivity to amiloride and its analogues while domains 4 and 7 participate in the Na^+^ and H^+^ transport. NHEs may form dimer or tetramer complexes through intermolecular interactions between transmembrane regions of the respective monomers [12, 33, 34].

### 1.3. NHE Regulation

**Figure 1:**
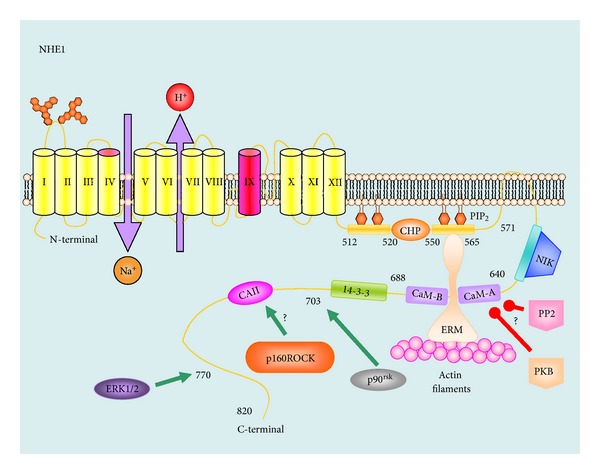
Model of transmembrane organization and regulation of the NHE1 exchanger. Transmembrane domains IV and IX (in red) are involved in the sensitivity of amiloride and its derivates. Numbers in the C-terminus domain indicate amino acid number in the structure. It is thought that intracellular H^+^ activates amino acids 445 and 446 in the transmembrane domain. Green arrows indicate sites of activation by different kinases while red arrows indicate sites of negative regulation by kinases or PP2. 14-3-3: 14-3-3 protein, CAII: carbonic anhydrase II, CaM-A and CaM-B: calcium-calmodulin A and B, CHP: calcineurin B homolog protein, ERK_1/2_: extracellular signal-related kinase, ERM: ezrin, radixin, and moesin, NIK: Nck-interacting kinase, p90^rsk^: ribosomal protein S6 kinase, p160ROCK: p160-Rho-associated kinase, PIP2: phosphatidylinositol 4,5-bisphosphate, PKB: protein kinase B, and PP2: protein phosphatase 2.

The NHE family is regulated by posttranslational modifications including protein kinase-mediated phosphorylation and by a number of signaling molecules including phosphatidylinositol-4,5-bisphosphate (PIP_2_), calcineurin homologous protein (CHP), ezrin, radixin, moesin (ERM), calmodulin, and carbonic anhydrase II. Mitogen-activated protein kinase (MAPK) signal transduction pathways are among the most widespread mechanisms of eukaryotic cell regulation. Mammalian MAPKs are activated by a wide variety of stimuli that include hormones, growth factors, inflammatory cytokines, osmotic shock, ischemic injury, and intracellular acidosis [35]. Upon activation, MAPKs phosphorylate NHE1. In particular, intracellular acidification leads to activation of the serine/threonine protein kinase Raf which then activates MEK (a MAPK kinase) that in turn activates extracellular signal-related kinase (ERK_1/2_) and ribosomal protein S6 kinase (p90^rsk^). ERK_1/2_ phosphorylates serine 770 and 771 while p90^rsk^ phosphorylates serine 703 of the NHE1 protein (Figure 1) [36–42]. Moreover, NHE1 is phosphorylated by p160-Rho-associated kinase (p160ROCK) [43] and Nck-interacting kinase (NIK) [44]. The mechanisms by which protein phosphorylation enhances H^+^/Na^+^ exchange are unclear. However, it has been suggested that phosphorylation facilitates binding of carbonic anhydrase II, which in turn catalyses the hydration of CO_2_ to form HCO_3_^−^ and H^+^ (Figure 1) [45]. NHE1 is also activated by calmodulin [46, 47], CHP1, CHP2 and tescalin (CHP3) [48–50]. Of note, the CHP-interacting region is flanked by two positively charged clusters that bind PIP_2_ *in vitro* which are important for NHE1 activity [51]. In addition, NHE1 binds to ERM cytoskeleton proteins which are important for signaling, cell migration, and apoptosis [52]. 

NHE1 is subject to inhibition. There is evidence that intracellular acidosis can negatively modulate NHE1 through phosphorylation by protein kinase B (PKB) [53] or dephosphorylation through protein phosphatase 2A (PP2A) [54]. This phosphorylation would interfere with Ca^2+^-calmodulin binding and could reduce the affinity for intracellular H^+^.

## 2. Role of NHE in Nociception

### 2.1. Inflammatory Pain

**Figure 2:**
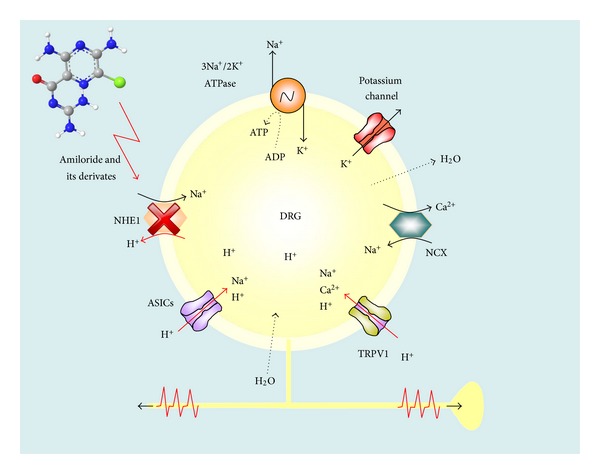
Role of NHE1 in intracellular pH regulation and nociception. In inflammatory pain, intracellular acidosis generated from tissue damage at the periphery, as well as from neurotransmitter release at the spinal cord, is counterbalanced by NHE1 contained in the nociceptive fibers. When amiloride or its derivates inhibit NHE1, an excess of intracellular hydrogen ions is accumulated in such a way that increases nociceptive fibers activity and consequently pain intensity. ASICs: acid-sensingion channels, DRG: dorsal root ganglia, NCX: Na^+^/Ca^2+^ exchanger, NHE1: Na^+^/H^+^ exchanger 1, and TRPV1: transient receptor potential cation channel subfamily *V* member 1.

The role of NHE in pain processing has been studied recently. Blockade of peripheral NHE with nonselective NHE inhibitors such as amiloride and 5-(N,N-dimethyl)amiloride (DMA) increases flinching behavior in the capsaicin, serotonin, and formalin tests. In addition peripheral injection of 5-(N-ethyl-N-isopropyl)amiloride (EIPA), a selective NHE1 inhibitor, also increases nociception in the same models [55]. These studies suggest that peripheral NHE1 is the main responsible for the actions of the peripheral NHE inhibitors. Furthermore, spinal blockade of NHE1 with selective NHE1 inhibitors EIPA and zoniporide increases flinching behavior induced by formalin [56]. In line with these studies, in the rat skin-nerve preparation, amiloride increased pH-induced nociceptor (C-fibers) spike discharge [57]. Amiloride enhanced both the duration and the magnitude of the response. Authors attributed this effect to the blockade of NHE. NHE1 mRNA and protein are found in the dorsal root ganglia and lumbar dorsal horn [55]. Taken together, data suggest that NHE1 plays an important role as an intracellular pH sensor and as a protective mechanism in nociceptive neurons in acute inflammatory pain states (Figure 2). In addition, it has been shown that blockade of peripheral and spinal NHE1 promotes but not maintains long-lasting bilateral secondary allodynia and hyperalgesia induced by formalin suggesting that NHE1 plays a role as a protective system in chronic pain as well [58]. Reinforcing this, NHE1 is downregulated from day 1 to 12 after formalin injection [58]. Similar results have been observed in biopsies from patients with ulcerative colitis and Crohn's disease that present an inflammatory process and abdominal pain [59, 60]. 

### 2.2. Neuropathic Pain

The role of NHE1 in neuropathic pain has been less studied. Systemic injection of amiloride attenuated chronic constriction injury- and vincristine-induced neuropathic pain [61]. Authors attributed the observed antinociceptive effects of amiloride to the inhibition of NHE with subsequent decrease in Ca^2+^ ions and oxidative stress. However, since they used doses of amiloride that also block acid sensing ion channels (ASICs) [62], it is likely that these effects may result from the blockade of ASICs instead of NHE. However, the final answer still needs confirmation.

Contrary to the results in inflammatory pain and in the skin-nerve preparation, other authors have reported that blockade of NHE1 by zoniporide reduces the amplitude of the compound action potential recorded from the dorsal root [63]. This study showed that blockade of NHE1 may reduce peripheral neuronal excitability by shifting fast Na^+^ channels into the inactivated state under physiological conditions. These actions may lead to antinociceptive effects. However, the same group has reported that continuous intravenous infusion of zoniporide to rats and dogs for up to 1 month, but not for 2-weeks, produced peripheral neuropathies (axonal degeneration), in the spinal cord (dorsal funiculus), dorsal roots, and dorsal root ganglia [64]. Thus, more research is need on this point to clarify the role of NHE1 in neuropathic pain. 

### 2.3. NHE in Nociceptive Neurons

NHE1 has been reported in dorsal root ganglia, dorsal spinal cord, and trigeminal neurons. NHE1 mRNA and protein expression are observed in dorsal root ganglia and dorsal spinal cord of rats [55]. Moreover, NHE5 protein is observed in spinal cord but not in dorsal root ganglia [55]. NHE1 is mainly expressed in the lamina I of the dorsal horn of the spinal cord and it colocalizes with peptide-rich sensory nerve fiber markers, substance *P*, and calcitonin gene-related peptide [56]. Others have found NHE1 in trigeminal ganglia [65] and colonic mucosa [59, 66]. Furthermore, NHE1 transcript has been found in human dorsal root ganglion [67, 68]. Data about the localization of NHE1 in neurons suggest that regulation of pHi may play a role in the nociceptive processing at peripheral and central sites (Figure 2). 

### 2.4. NHE in Schwann Cells

NHE1 has been found in primary cultures of Schwann cells from rat sciatic nerve [69]. Authors found that NHE was moderately active at steady-state pHi. More recently, NHE3 has been found in Schwann cells on the laryngeal nerve [70]. Nerve fibers and nerve cell bodies of Schwann cells and satellite cells were surrounded by both proteins. It is likely that, as in other cells, NHE plays a role in Schwann cells regulating pHi. However, it has been reported that NHE may have a role in proliferation of Schwann cells as inhibition of NHE after addition of a mitogen significantly reduced the degree of mitosis [71]. 

### 2.5. NHE in Microglia

NHE1 is expressed in resting microglia [72]. Pharmacological inhibition of NHE1 activity acidifies primary or immortalized M4T.4 microglia in resting conditions and blockades pHi recovery capacity after experimental acidification [72–74]. These data suggest that NHE1 plays a key role in maintaining pHi in resting conditions and extruding H^+^ after acidosis in microglia. Activation of microglia by lipopolysaccharide does not change the expression of NHE1 but increases the activity to maintain pHi. In addition, lipopolysaccharide increases the production of the superoxide radical (O_2_^•−^) in microglia while inhibition of NHE1 reduces microglial activation and proinflammatory response. These data suggest that NHE1 participates in the generation of O_2_^•−^ through maintaining H^+^ homeostasis, thereby allowing for sustained NADPH oxidase complex activation in activated microglia [72]. Free radicals can subsequently lead to release of cytotoxic proinflammatory cytokines. Since microglial activation and release of cytokines have been associated with inflammatory and neuropathic pain [75, 76], it has been suggested that NHE1 may be one of the mechanisms to increase microglial activity and sustain neuropathic pain [63, 64]. 

### 2.6. NHE in Astrocytes

Astrocytes play an important role throughout the central nervous system among others regulating pH [13]. Injury or stress to the central nervous system activates astrocytes, which then display an altered morphology and protein expression [77]. NHE1 protein has been found in astrocytes [78–81]. It seems that NHE1 is moderately active in basal conditions, but it can be activated by phosphorylation through tyrosine kinase (TK), ERK_1/2_, and p90^rsk^, in astrocytes [78, 82, 83] further promoting extrusion of acid. Other substances like tumor necrosis factor-alpha (TNF*α*), interferon-*γ*, interleukin-1 beta (IL-1*β*), and hydrogen sulfide (H_2_S) also produce intracellular acidification and activation of NHE in astrocytes. In contrast, cyclic GMP-inducing C-type natriuretic peptide and cyclic GMP inhibit NHE in astrocytes [84]. 

### 2.7. Role of NHE1 in Inflammatory Pain in Humans

Inflammatory bowel diseases such as Crohn's disease and ulcerative colitis have been associated with defects in homeostasis of cations as revealed by altered expression of several cation transporters [59, 85]. It is thought that these defects may be responsible for motility dysfunction, diarrhea, and pain commonly seen in patients with this type of diseases. NHE1 plays an important role in cation homeostasis of the gastrointestinal tract [27]. There are consistent reports that NHE1 is reduced in ulcerative colitis and Crohn's disease in humans [59, 60]. Authors suggest that this reduced expression may compromise recovery of acidic pHi, and thus it may contribute to tissue necrosis and probably to pain [59, 66]. However, on the bases of the present data, we cannot discharge that other mechanisms might be contributing to produce the characteristic symptoms of the Crohn's disease and ulcerative colitis.

On the contrary, NHE inhibition of human gut epithelial cells suppressed interleukin-8 production and activation of the p42/p44 mitogen-activated protein kinase and nuclear factor-kappaB. Furthermore, NHE inhibition ameliorated the course of inflammatory bowel disease in dextran sulfate-treated mice [86]. In support of this, NHE inhibitors may produce an anti-inflammatory effect by inhibiting the production of PGE_2_ and the increase in COX-2 protein levels [87]. Differences could be due to the experimental approach used. However, more studies are needed in order to clarify this issue.

### 2.8. Perspectives and Conclusion

The role of NHE1 in nociception has recently been discovered. Data suggests that NHE1 plays a protective role in acute and chronic inflammatory pain. However, the role of NHE1 in neuropathic pain is controversial. Since NHE1 inhibitors produce an increase of inflammatory pain, the study of NHE1 inhibitors in neuropathic pain is difficult because models of neuropathic pain do not allow getting a graded level of allodynia in such way that blockade of NHE1 would allow assessing an increase in tactile allodynia. The development of NHE1 activators could help to solve the problem. The results observed in the acute and chronic model of inflammatory pain induced by formalin should be corroborated in other models of inflammatory pain. Particularly, the use of models related to chronic inflammatory conditions, in which acidification is a common feature, such as the injection of complete Freund's adjuvant (CFA), monoiodoacetate (MIA), or uric acid, is recommended. The use of knock-out mice as well as interference RNA directed against NHE1 and other members of the family would be helpful to delineate the participation of these proteins in the modulation of pain. The wide distribution of NHE1 could represent a challenge for drug development. Besides nociceptive neurons, NHE1 is found in heart and brain. Thus, activation of NHE1 may lead to side effects in those sites. However, the integrated study of the pHi regulation involving NHE1 will definitely produce the basis to understand how nociceptive sensory neurons function in presence of the acidic conditions. 

## Figures and Tables

**Table 1:** Characteristics of the Na^+^/H^+^ exchanger (NHE) family.

Common name (gene)	Distribution	Membrane Localization	Function	Pathophysiology
NHE1 (SLC9A1)	Ubiquitous	Plasma membrane	Cytosolic pH, cell volume, fluid secretion, cell shape, proliferation, migration	Ataxia, seizures, ischemia, reduced parotid gland secretion, pain
NHE2 (SLC9A2)	Several tissues	Plasma membrane	Fluid secretion	Loss of acid secretion, reduced parotid gland secretion
NHE3 (SLC9A3)	Kidney, intestines	Plasma membrane	Reabsorption of Na^+^ and HCO_3_^−^	Diarrhea, tubular proteinuria, hypertension
NHE4 (SLC9A4)	Stomach Kidney	Plasma membrane	Cytosolic pH, fluid secretion	Impaired gastric acid secretion
NHE5 (SLC9A5)	Brain, testis, spleen, and skeletal muscle	Plasma membrane	Cytosolic pH	Pain
NHE6 (SLC9A6)	Ubiquitous	Endosomes	Organellar pH	X-linked mental retardation, epilepsy, ataxia
NHE7 (SLC9A7)	Ubiquitous	Endosomes	Organellar pH	Unknown
NHE8 (SLC9A8)	Ubiquitous	Endomembranes	Organellar pH	Unknown
NHE9 (SLC9A9)	Ubiquitous	Endosomes	Organellar pH	Attention-deficit hyperactivity disorder, autism-spectrum disorder
